# A novel lineage restricted, pericyte-like cell line isolated from human embryonic stem cells

**DOI:** 10.1038/srep24403

**Published:** 2016-04-25

**Authors:** Midori Greenwood-Goodwin, Jiwei Yang, Mohammad Hassanipour, David Larocca

**Affiliations:** 1 ReCyte Therapeutics, Inc., Alameda, CA 94501, USA; 2 StemCells, Inc., Newark, CA 94560, USA

## Abstract

Pericytes (PCs) are endothelium-associated cells that play an important role in normal vascular function and maintenance. We developed a method comparable to GMP quality protocols for deriving self-renewing perivascular progenitors from the human embryonic stem cell (hESC), line ESI-017. We identified a highly scalable, perivascular progenitor cell line that we termed PC-A, which expressed surface markers common to mesenchymal stromal cells. PC-A cells were not osteogenic or adipogenic under standard differentiation conditions and showed minimal angiogenic support function *in vitro*. PC-A cells were capable of further differentiation to perivascular progenitors with limited differentiation capacity, having osteogenic potential (PC-O) or angiogenic support function (PC-M), while lacking adipogenic potential. Importantly, PC-M cells expressed surface markers associated with pericytes. Moreover, PC-M cells had pericyte-like functionality being capable of co-localizing with human umbilical vein endothelial cells (HUVECs) and enhancing tube stability up to 6 days *in vitro.* We have thus identified a self-renewing perivascular progenitor cell line that lacks osteogenic, adipogenic and angiogenic potential but is capable of differentiation toward progenitor cell lines with either osteogenic potential or pericyte-like angiogenic function. The hESC-derived perivascular progenitors described here have potential applications in vascular research, drug development and cell therapy.

Pericytes (PCs) are integral to the development, maturation and stabilization of vasculature. PCs wrap around the endothelial cells (ECs) to provide scaffolding support and regulate EC behavior, such as the formation of endothelial cell-cell junctions. PCs also regulate EC migration, differentiation and stabilization through pericyte-EC direct cell contacts and paracrine signaling pathways^1^. Furthermore, PCs may function as multipotent mesenchymal stromal cells (MSCs) or perivascular stromal cells (PSCs) serving as a source of repair cells that are activated following injury. A lack of functional PCs is associated with a variety of pathologic conditions, including neurodegenerative disorders, ischemic disorders and diabetic retinopathy^2^. Preclinical studies indicate therapeutic potential of PCs for regenerative treatments for a multitude of disorders, including bone defects, limb ischemia, ischemic heart disease, muscular dystrophy and retinal vasculopathy^3^,^4^,^5^,^6^,^7^. Translation of pericyte research to the clinic will require a scalable, well defined cell source. The use of primary cells for regenerative medicine is limited because of batch to batch variation, cell heterogeneity, low replicative capacity and loss of function in culture. Moreover, the use of autologous stem cells for therapy could be limited by the age or health status of the patient. For example, MSCs lose both osteogenic and vascular support function with aging^8^. Derivation of PCs from human embryonic stem cell (hESC) lines offers the possibility of a renewable and scalable source of uniform cells for research and development of regenerative therapies.

Previous studies have identified primary pericytes and human pluripotent stem cell (hPSC) derived pericyte-like cells with both angiogenic support function and MSC-like multi-lineage potential^4^,^9^. However, recent mouse studies suggest that specialized subtypes of pericytes may exist with more restricted lineage potential^10^. Here we demonstrate the derivation of 3 distinct progenitor cell types from the GMP compatible hESC line, ESI-017^11^. Using a modified endothelial cell derivation protocol, we first derived a self-renewing perivascular progenitor cell type we termed PC-A. PC-A cells expressed multipotent stem cell markers like CD133 and CD34, but lacked osteogenic or adipogenic potential and angiogenic support function. Further directed differentiation of PC-A cells resulted in the generation of 2 distinct perivascular progenitor cell types; one with osteogenic potential (PC-O) and a second with pericyte-like angiogenic support function (PC-M). Both of the PC-A derived cell types failed to differentiate to adipocytes under conditions that successfully differentiated bone marrow derived mesenchymal stromal cells (BM-MSC) to adipocytes. We have thus derived a novel scalable progenitor cell from hESCs that can be used as a source of at least 2 distinct lineage restricted progenitor cell types.

We established the identity of all 3 progenitor cell types by surface marker expression. Notably, the pericyte-like cell type, PC-M cells, expressed CD146 and CD105, suggesting that these cells may have angiogenic support function similar to PCs and MSC sub-populations identified *in vivo*^12^. Using a modified *in vitro* Matrigel^TM^ tube formation assay, we found that PC-M cells have angiogenic support function similar to or greater than primary placental pericytes (Pl-PCs) and BM-MSCs. Specifically, PC-M cells co-localized with human umbilical vein endothelial cells (HUVECs) and provided superior tube stabilization. We have thus derived a scalable, pericyte-like cell, PC-M, with angiogenic support function characteristic of pericytes. PC-M cells are a novel, well defined and highly expandable cell type with the potential to be further developed for improved *in vitro* angiogenesis assays, drug screening, and cell therapy applications.

## Results

### Derivation of self-renewing hESC-derived perivascular progenitors with stable morphology and high scalability

**Figure 1:**
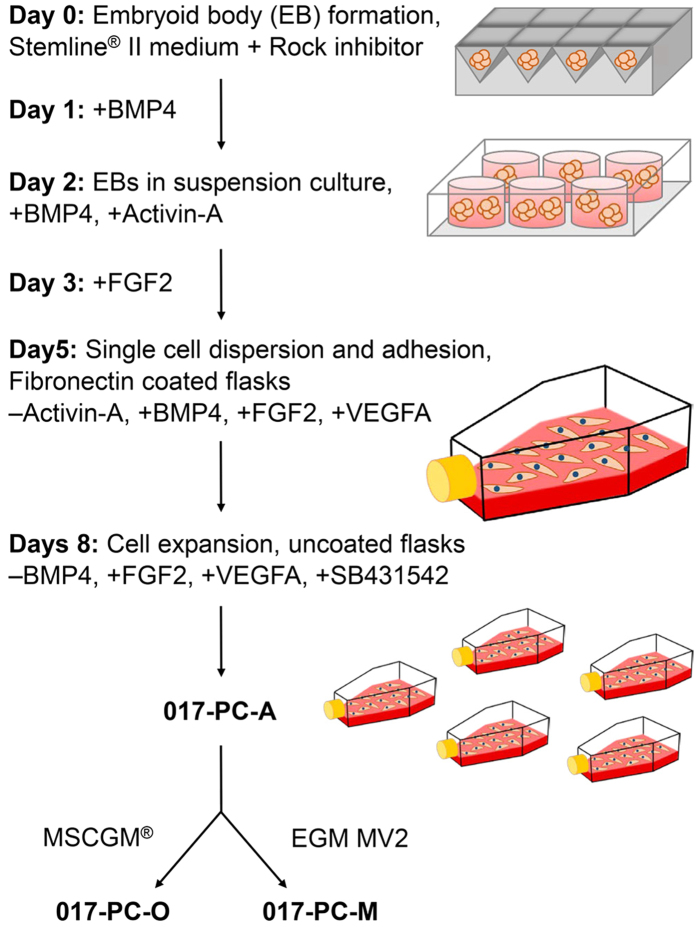
Schematic representation of culture conditions for generation of hESC-derived perivascular progenitors. The 3 progenitor cell lines, 017-PC-A, 017-PC-O and 017-PC-M were generated following culture and/or expansion in modified Stemline^TM^ II Media, Mesenchymal Stem Cell Growth Media (MSCGM^TM^) or Endothelial Cell Growth Medium MV2 (EGM-MV2), respectively.

Multiple progenitor cell lines were derived from the human embryonic stem cell (hESC) line ESI-017 using a modified protocol previously established for the generation of endothelial progenitor cells^13^. We seeded ESI-017 cells at multiple densities to generate embryoid-bodies (EBs) in AggreWell^TM^ plates and then transferred the EBs as single cell suspensions to adherent culture conditions, screening for differences in cell morphology (Fig. 1). The resulting cell cultures showed significantly different cell morphologies as a function of initial cell seeding density during EB generation. Adherent cell cultures derived from EBs formed at low cell seeding densities had endothelial-like progenitor cell morphology, whereas those formed at high cell seeding densities had a mesenchymal-like cell morphology (Supplementary Fig. S1). We hypothesized that these mesenchymal-like cells were perivascular progenitors and termed them 017-PC-A (PC-A) cells. PC-A cells were further differentiated toward 2 distinct cell lines based on cell morphology and scalability. 017-PC-M (PC-M) cells, derived in endothelial cell growth medium (EGM-MV2) had a morphology similar to PC-A cells. In contrast, 017-PC-O (PC-O) cells, derived in mesenchymal stem cell growth media (MSC-GM) had a morphology similar to primary MSCs (Supplementary Fig. S2).

**Figure 2:**
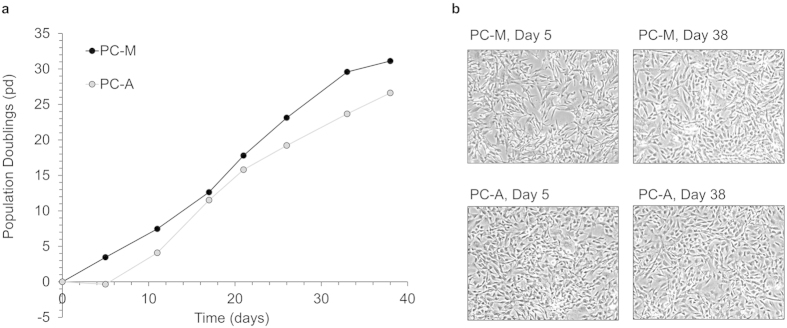
Cell growth rates, expansion and morphology of hESC-derived perivascular progenitors over-time. (**a**) Both hESC-derived (017-) PC-A and PC-M cells are highly scalable with no significant decrease in cell doubling rates from day 0 (passage 4) to day 38 (passage 11). (**b**) hESC-derived (017-) PC-A and PC-M cells, have similar morphology between cell types and over multiple passages.

PC-A and PC-M cells were highly scalable; PC-A and PC-M cells underwent up to 31 population doublings over 38 days of culture and 7 passaging events without a significant decrease in growth rate (Fig. 2a). PC-A and PC-M cells had similar population doubling times. PC-A cells had an average population doubling time of 0.66 ± 0.30 pd/day. PC-M cells had an average population doubling time of 0.80 ± 0.09 pd/day. In contrast, PC-O cells had a significantly reduced growth rate of 0.15 ± 0.05 pd/day and therefore had limited expansion capacity (data not shown). In addition to high scalability, PC-A and PC-M cells show stable cell morphology following 38 days of cell culture (Fig. 2b).

### hESC-derived perivascular progenitors express mesenchymal and perivascular markers

**Figure 3:**
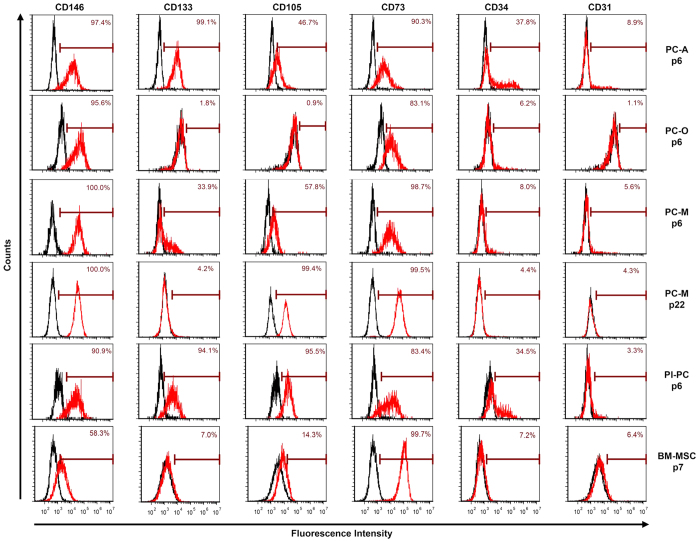
Immunophenotyping of hESC-derived perivascular progenitors and primary perivascular cells. Representative flow cytometry analysis of the following sets of markers: (1) pericyte associated cell surface marker: CD146, (2) multipotent stem cell surface marker CD133, (3) vascular endothelium/pericyte associated marker: CD105, (4) mesenchymal stromal cell (MSC) cell surface markers: CD73, and (5) endothelial cell surface markers: CD34, CD31. The histograms shown were gated on live cells using forward and side scatter profiles. The percentage of positive cells for specified markers (red) is shown here compared with isotype controls (black).

We assessed the 3 hESC-derived perivascular progenitors for cell surface markers associated with mesenchymal stromal cells, pericytes and endothelial cells (Fig. 3). All cells were assayed by flow cytometry following extended passage in their respective expansion or derivation medium (Fig. 1, Days 8+). We found that all 3 perivascular progenitors were positive for CD146 and CD73 (83–100%). Furthermore, all 3 perivascular progenitors showed low or no expression of CD31 (<10%). PC-A cells, but not PC-M or PC-O cells showed intermediate expression of CD34 (38%). Interestingly, PC-O cells were negative for CD133, while PC-A cells were positive for CD133 (>98%) and PC-M cells showed intermediate expression (p6, 34%). Further expansion of PC-M cells resulted in loss of CD133 (p22, 4%). PC-M cells were also negative for pluripotency markers Tra-1-60 and Oct-4 (data not shown).

Flow cytometry analysis of PC-M cells at multiple passages was used to further establish cell identity. Expression of CD105, PDGFRβ and NG-2 markers in PC-M cells changed over multiple passaging events and PC-M cell expansion. We found that PC-M cells were initially positive for pericyte markers, PDGFRβ (40–50%) and NG-2 (10–20%) at passage 3 (p3, data not shown), but rapidly lost expression of both markers (p6, Supplementary Fig. S3). In contrast, PC-M cells showed an increase in expression of CD105 from intermediate to late passages (p6, 58%; p22, 99%, Fig. 3). Notably, late passage PC-M cells displayed a similar surface marker profile to pericytes derived from induced pluripotent stem cells^14^,^15^.

To further establish cell identity of all 3 perivascular progenitors, we expanded placental pericytes (Pl-PCs) and bone marrow derived MSCs (BM-MSCs) and assayed Pl-PCs (p6) and BM-MSCs (p7) for identical surface markers (Fig. 3). Pl-PCs highly expressed CD146, CD133, CD105 and CD73 (>80%), showed intermediate expression of CD34 (35%), relatively low expression of PDGFRβ and NG2 (10–30%, Supplementary Fig. S3) and did not express CD31 (<5%). BM-MSCs were positive for CD73 (>99%), showed intermediate expression of CD146 (58%) and low expression of CD133, CD105, CD34 and CD31 (5–15%).

### hESC-derived perivascular progenitors have restricted differentiation potential

**Figure 4:**
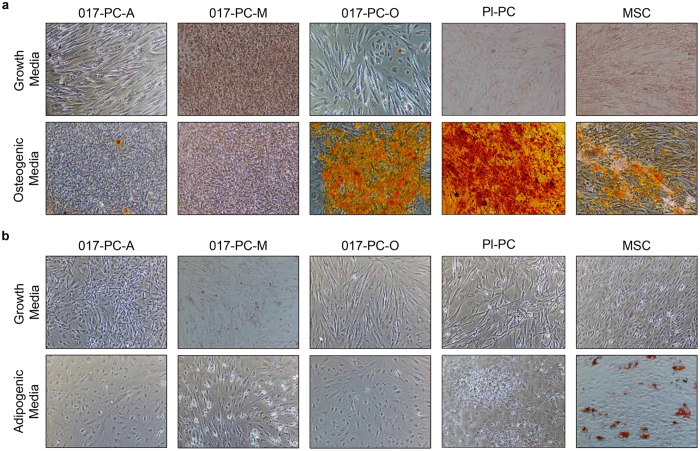
Osteogenic and adipogenic induction of hESC-derived perivascular progenitors and primary perivascular cells. (**a**) All cells were cultured in either growth media (top row) or osteogenic induction media (bottom row). Cells were fixed and stained with Alizarin Red S to qualify the extent of calcium deposits at day 21 of culture. Qualitatively, extensive Alizarin Red S suggests that 017-PC-O cells, Pl-PCs and MSCs have osteogenic differentiation potential. (**b**) All cells were cultured in either growth media (top row) or adipogenic induction media (bottom row). Cells were fixed and stained with Oil Red O to assess lipid accumulation at day 14 of culture. Only MSCs stained positive for Oil Red O and have adipogenic differentiation potential.

To investigate the functional multipotency of all 3 hESC-derived perivascular progenitors, we examined the capability of these cells to differentiate into osteoblasts and adipocytes *in vitro*. Previous studies have demonstrated that MSCs and PCs are capable of differentiation to both osteoblasts and adipocytes. Using osteogenic or adipogenic differentiation media, all 3 hESC-derived perivascular progenitors were assayed for differentiation potential (Fig. 4). For reference, primary Pl-PCs and BM-MSCs were similarly assayed (Fig. 4). The extent of calcium-rich mineralization of the cell matrix was assessed using Alizarin Red S staining after 21 days of culture in osteogenic medium (Fig. 4a). PC-A and PC-M cells were not osteogenic having little or no Alizarin Red staining under these conditions. However, PC-A cells, but not PC-M cells, showed significantly altered cell morphology in osteogenic differentiation media compared with growth media (Fig. 4a). PC-O cells, Pl-PCs and BM-MSCs displayed extensive calcification and demonstrated osteogenic differentiation potential, with Pl-PCs exhibiting the most extensive calcification. After 14 days of culture in adipogenic media the extent of lipid droplet formation was assessed by Oil Red O staining (Fig. 4b). Only BM-MSCs stained positive for accumulation of lipid droplets. Therefore, only BM-MSCs demonstrated both osteogenic and adipogenic potential.

### PC-M cells stabilize endothelial cells and resulting vasculogenic tube networks *in vitro*

**Figure 5:**
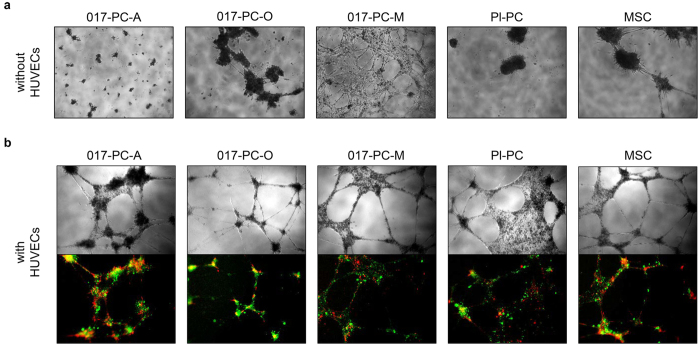
Tube formation by hESC-derived perivascular progenitors and primary perivascular cells. (**a**) hESC-derived perivascular progenitors and primary cells were cultured on growth factor reduced (GFR)-Matrigel^®^ and imaged at 24 hours to monitor the extent of tube assembly. Cells were seeded at 25,000 cells per well using 96 well plate format. (**b**) hESC-derived perivascular progenitors and primary cells (red) were co-cultured with umbilical vein endothelial cells (HUVECs, green) on GFR-Matrigel and imaged at 24 hours to monitor the extent of tube assembly. Co-cultures were seeded with 40,000 HUVECs and 2,000 hESC-derived perivascular progenitors or primary cells per well using 96 well plate format. All cultures were treated identically, without media exchange or the addition of exogenous growth factors. Fluorescent images show the presence of both HUVECs and test cells throughout the tube network, and extensive co-localization of test cells and HUVECs within dense cell clusters (yellow).

We examined the ability of all 3 hESC-derived perivascular progenitors to support angiogenesis by seeding the cells on growth factor reduced-Matrigel in monoculture and co-culture with human umbilical vein endothelial cells (HUVECs) *in vitro*. In monocultures, we assessed the ability of these cells to form tube networks. At 1 day of monoculture, only PC-M cells formed tube networks with extensive branching between flat cell sheets (Fig. 5a). PC-O cells and BM-MSCs formed tube-like structures, but showed large, dense cell clusters and less branching. PC-A cells formed small cell clusters with minimal branching. Pl-PCs formed large cell clusters with no observable tube-like structures or branching. We next assessed the ability of hESC-derived perivascular progenitors or primary cells to stabilize HUVEC tube networks formed by HUVECs in co-cultures. At 1 day of co-culture, tube networks were observed in all co-cultures (Fig. 5b). The representative images of the resulting tube networks show that co-cultures containing variable test cells, have variable tube thickness, branching and cell clustering at branch points (Fig. 5b). The total tube network length, including edges of cell sheets, was not significantly different across co-culture conditions and compared with HUVECs in monoculture (Supplementary Fig. S4). However, the average total branching length was highest for co-cultures containing PC-M cells. Notably, PC-M cells were localized along the outside of tube-like structures and not in dense cell clusters (Fig. 5b), mimicking the architecture of pericyte cells *in vivo,* where pericytes wrap around endothelial cells^1^. Overall, PC-M cells co-cultured with HUVECs have minimal cell clustering and thicker (denser) tube structures compared with all other cell types. In contrast, PC-A cells, PC-O cells and BM-MSCs co-cultured with HUVECs show co-localization with HUVECs into larger cell clusters and thinner (less dense) tube structures. Lastly, Pl-PCs co-cultured with HUVECs show extensive cell sheet formation and tube structures of variable thickness. Qualitatively, the 3 hESC-derived progenitors and primary cells, each have distinct effects on the macrostructure of tube networks formed by HUVECs.

**Figure 6:**
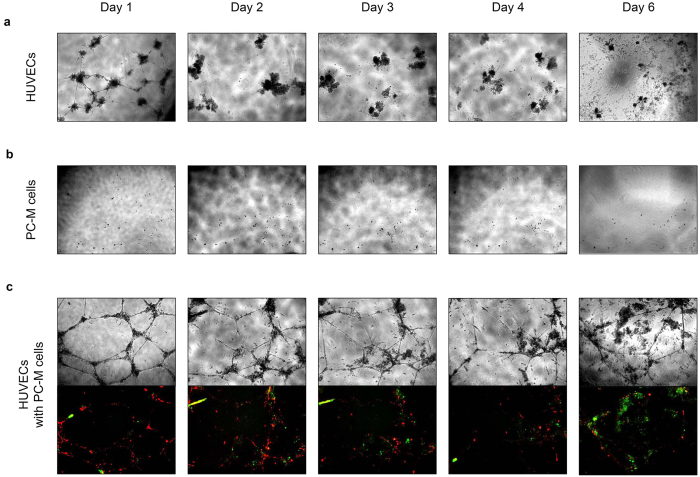
hESC-derived perivascular progenitor, 017-PC-M, supports vasculogenic tube formation and stability in HUVEC co-cultures. (**a**) HUVECs were seeded at a density of 40,000 cells per well using 96 well plate format. In monoculture, HUVECs assemble into tube-like structures and larger networks by 4 hours. Tube networks do not persist after day 1. (**b**) (017-)PC-M cells were seeded at a density of 2,000 cells per well using 96 well plate format. In monoculture, PC-M cells at low seeding densities do not form tube networks or significantly proliferate over 6 days. (**c**) Cells were seeded at 42,000 cells per well using 96 well plate format at a ratio of 20:1 HUVECs to PC-M cells (40,000 HUVECs to 2,000 PC-M cells). All cultures were treated identically, without media exchange or the addition of exogenous growth factors. In co-culture, tube networks assembled by HUVECs with 017-PC-M cells are more extensive and thicker. Tube networks formed in co-cultures undergo minimal degradation and persist for up to 6 days.

We hypothesized that PC-M cells may further stabilize endothelial tube networks *in vitro*, a hallmark of pericyte function^12^,^16^. We found that increasing the ratio of HUVECs to PC-M cells significantly improves the initial tube network formation, as well as long-term stability (Supplementary Fig. S5). When seeded at a ratio of 20:1, HUVECs to PC-M cells, PC-M cells improve tube network formation and stability over the course of 6 days (Fig. 6). In monoculture, HUVECs formed a complete tube network within 4–8 hours. This network remained intact at 1 day and began to degrade by 2 days (Fig. 6a), after which the HUVECs further dispersed and were unable to establish a tube network over the course of 6 days. In monoculture, PC-M cells form small clusters or remain as isolated cells over the course of 6 days when seeded at a low number correlating with the number of PC-M cells in co-culture (Fig. 6b). In co-culture, HUVECs and PC-M cells formed an extensive tube network, showing similar branching to HUVEC monocultures, but longer and thicker or denser tube-like structures (Fig. 6c). Minimal degradation of tube structures is observed in co-culture and the presence of an interconnected tube network persisted for at least 6 days without media exchange or the addition of exogenous growth factors (Fig. 6c). Only PC-M cells provided long-term tube stability up to 6 days compared with PC-A cells, PC-O cells and primary cells (Supplementary Fig. S6). These results demonstrate that PC-M cells are an angiogenic support cell type, consistent with pericyte cell functionality *in vitro.*

## Discussion

Perivascular cells from primary cell sources have significant heterogeneities between and within various cell types, such as mesenchymal stromal cells (MSCs), vascular smooth muscle cells (vSMCs) and PCs^15^. Although these cell types may have similar functionality *in vivo*, primary cell sources are limited by their expandability and activity *in vitro*, limiting the use of the cells in basic and translational research. To address the limitations of primary cell types, we have developed a method for the derivation of perivascular progenitors from hESCs. We hypothesized that modification of a method for deriving endothelial progenitor cells (EPCs) from hESCs could yield highly expandable, stable vascular support cell types, such as pericytes (13). We found that increasing the initial seeding density during embryoid body (EB) formation supported the derivation of a novel, perivascular progenitor cell type, termed PC-A. Further expansion of PC-A cells in mesenchymal cell culture medium or endothelial cell culture medium, was used to derive 2 additional perivascular progenitor cells, termed PC-O and PC-M, respectively. We attempted continuous culture and expansion of all 3 perivascular progenitors in their respective cell culture medium. We showed that PC-A cells and PC-M cells were scalable, therefore these cell lines may be suitable for industrial scale production for research and clinical development. In contrast, PC-O cells were not scalable, having a low population doubling rate. In the present study, we explored only 2 cell culture variables–cell seeding density during EB formation and cell culture medium. Therefore, it is probable that alternative cell density and expansion conditions exist which may yield additional cell types. Furthermore, the derivation and culture methods described here may be further modified to support production of additional cell types, including clonal cell populations of PC-A and/or PC-M cells.

**Figure 7:**
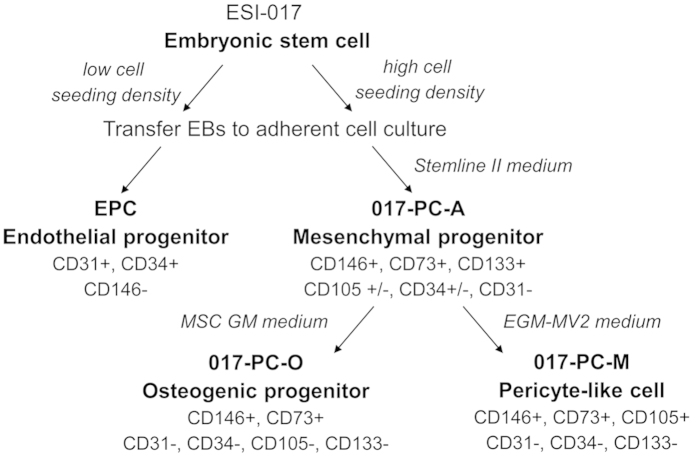
hESC based derivation and characterization of perivascular progenitors. A common precursor cell population (017-PC-A) was generated by screening multiple embryoid body (EB) seeding densities and culture media conditions. 017-PC-A cells can be further differentiated toward pericyte-like cells (017-PC-M) capable of endothelial support function or a third cell type (017-PC-O) capable of osteogenic differentiation. Both 017-PC-A and 017-PC-M cells are scalable, self-renewing progenitors. All 3 hESC-derived perivascular progenitors have restricted differentiation potential and do not differentiate toward adipocytes.

We showed that all 3 hESC-derived perivascular progenitors have unique expression of multiple cell surface markers, with similar expression observed only for surface markers CD146 and CD73 (Figs. 3 and 7). Moreover, all 3 cell types demonstrated restricted mesenchymal lineage potential (Fig. 4). These results suggest that all 3 cell lines are different perivascular cell subtypes, each having a distinct differentiation potential^10^. For example, only PC-A cells were positive for CD133, suggesting that these cells are an early, progenitor cell with high proliferation and differentiation potential^18^. However, we found that PC-A and PC-M cells were unable to differentiate toward osteoblasts or adipocytes, whereas with further differentiation PC-A derived PC-O cells were able to differentiate toward osteoblasts, but not adipocytes. The lack of adipocyte differentiation potential of PC-A and PC-M cells suggests that these cells are immature stem cell types similar to fetal mesenchymal stromal cells, which also lack adipogenic potential^19^,^20^. Both PC-A and PC-M cells highly expressed CD146, a cell-adhesion molecule actively involved in angiogenesis^21^. However, only PC-M cells showed similar trends of marker expression as primary pericytes such as high expression of CD105 and low expression of CD133 and CD34, suggesting that PC-M cells are pericyte-like cells with angiogenic support function^12^,^22^. We propose that PC-A and PC-M cells are 2 unique subsets of perivascular cells with PC-A being a more primitive self-replicating progenitor. Recently, 2 subsets of pericytes with and without angiogenic function *in vitro* and *in vivo* were identified in mice. Notably, the pro-angiogenic pericyte subset identified in mice did not undergo adipogenic differentiation *in vitro*^10^.

Expression of pericyte markers is highly dynamic *in vitro* and *in vivo. In vivo,* pericyte-specific markers are known to vary across cell developmental stages and in various tissue types, such that subsets of pericyte cells have different expression of markers, including PDGFRβ and NG2^1^. *In vitro,* these same makers may also vary as a result of *in vitro* culturing. Further immunophenotyping of PC-M cells showed that these cells rapidly lost expression of 2 pericyte markers, PDGFRβ and NG2, during *in vitro* expansion. Although PDGFRβ and NG2 are associated with adult stem and progenitor cells, particularly from brain vasculature, the loss of these specific markers in PC-M cells during expansion culture does not preclude these cells from being identified as pericyte-like cells. Expression of CD105 increased following expansion of PC-M cells. The upregulation of CD105, which is upregulated during hypoxia and highly expressed in other angiogenic cell types, suggests that PC-M cells might be angiogenic or function as angiogenic support cells^23^. After expansion, PC-M cells were positive for CD73 and lost expression of CD133 (<5%) suggesting PC-M cells lack hematopoietic stem cells or residual undifferentiated hESCs. Importantly, the immunophenotype of PC-M cells at early and late passages showed that these cells were positive for CD146 and negative for CD34 (Fig. 3). Previously, isolation of CD146(+)/CD34(−) cells correlated with angiogenic support activity in pericyte cells from multiple tissues^12^. The stable expression of these markers led us to hypothesize that these were pericyte-like cells capable of supporting and stabilizing angiogenesis with high scalability needed for clinical applications.

The formation of functional and stable blood vessels *in vivo* depends on both endothelial cells and perivascular cells, including pericytes^1^. In the absence of definitive markers, pericytes can be identified functionally *in vitro* by their ability to co-localize with endothelial cells and stabilize tube network formation^12^. Vasculogenic tube assembly by endothelial cells in monoculture are unstable, with tube networks degrading after 1 day unless supported by a secondary cell type. In the present study, the second cell type provided is the hESC-derived perivascular progenitor cell, PC-M. PC-M cells in monoculture demonstrated the ability to form an independent tube network. Similar to endothelial cell tube networks in monoculture, PC-M tube networks in monoculture were unstable, with tube networks degrading after 2 days. In co-culture, PC-M cells demonstrated good angiogenic support function, stabilizing vasculogenic tube assembly by HUVECs. PC-O cells or BM-MSCs also supported stable tube formation but these were less stable than PC-M cell co-cultures. In contrast, co-cultures of HUVECs with PC-A cells or Pl-PCs were not stable (Fig. S6). The greater *in vitro* tube stabilization by PC-M cells might be a result of their earlier developmental status. Both HUVECs and PC-M cells were identified along the length of tube structures throughout the duration of the experiment (Fig. 6). These results indicate that the *in vitro* model recapitulates the direct cell to cell contact that is important role for stabilizing tube networks *in vivo*. Overall, these results are consistent with a model of vascular morphogenesis wherein pericyte migration, tube assembly and/or recruitment to existing tube networks is essential for the stable formation of vasculogenic tube assembly^24^.

In summary, we have developed a process for deriving novel perivascular progenitor cell line, PC-A, from hESCs (Fig. 7). PC-A cells can further be differentiated toward PC-O cells, which are osteogenic, or PC-M cells, which are not osteogenic but show significant *in vitro* angiogenic support function being capable of stabilizing HUVEC tube formation (Table 1). The methods used here may be readily scaled for basic research and clinical translation, since the source cell line, ESI-017, and culture conditions are comparable to GMP quality protocols. Notably, PC-A cells were expanded in serum-free medium prior to further differentiation. Both cell derivatives presented here have potential applications in cell therapy. PC-O cells could potentially be useful for developmental research, disease modeling, and clinical applications for bone repair and osteoporosis because of their osteogenic lineage restriction. PC-M cells may be useful for angiogenesis research, pro-angiogenic and anti-angiogenic drug screening, disease modeling and clinical applications requiring vascular support function but lacking other mesenchymal differentiation capacities. Further studies aimed at understanding the angiogenic potential of these cells *in vivo* using animal models of ischemic repair will be essential for establishing their potential in pro-angiogenic therapies.

## Materials and Methods

### Derivation of perivascular progenitors from human embryonic stem cells (hESCs)

Prior to derivation protocol, NIH-registered hESC line ESI-017 was expanded on growth factor reduced Matrigel^TM^ (Corning Life Sciences) in mTeSR^TM^-1 medium (Stemcell Technologies). On day 0, start of derivation, ESI-017 cells were detached using Accutase^TM^ (Life Technologies), pelleted and re-suspended into Stemline^TM^ II hematopoietic stem cell expansion medium (Stemline II; Sigma) supplemented with 10 μM Rock inhibitor, Y-27632 (Stemcell Technologies). Cells were seeded at 4,000 cells per microwell on an AggreWell^TM^ 400 plate (Stemcell Technologies). On day 1, Rock Inhibitor was withdrawn and the cell culture media was supplemented with 20 ng/mL bone morphogenetic protein 4 (BMP-4; HumanZyme). On day 2, uniform embryoid bodies (EBs) within individual microwells were transferred to an UltraLow Attachment 6-well plate (Corning Life Sciences) for EB suspension culture; cell culture media was further supplemented with 10 ng/mL Activin-A (HumanZyme). On day 3, cell culture media was further supplemented with 8 ng/mL basic fibroblast growth factor (FGF-2; HumanZyme). On day 5, the EBs were digested with Accutase and cells dispersed onto a fibronectin-coated T-150 flask for adherent cell culture; Activin-A was withdrawn and cell culture media was further supplemented 25 ng/mL vascular endothelial growth factor A (VEGF-A; HumanZyme). On day 8, cells were passaged and seeded onto uncoated T-150 or T-225 flasks; BMP-4 was withdrawn and cell culture media was further supplemented with 10 μM TGFβ signaling inhibitor SB431542 (Cayman Chemical). On day 11, the cells were harvested and aliquots were frozen in medium containing 10% DMSO and stored in liquid nitrogen. Cells were thawed with >90% recovery. As detailed above, all supplement concentrations were kept constant until the supplement was withdrawn.

### Perivascular progenitor cell culture and expansion

Perivascular progenitors were thawed and seeded at a density of 7.5–10 × 10^4^ cells per cm^2^ into T-75 or T-150 flasks. Cells were cultured and expanded in 1 of 3 media conditions. (1) Cells expanded in Stemline II media containing 8 ng/mL FGF-2, 25 ng/mL VEGF-A, and 10 μM SB431542 are termed 017-PC-A. (2) Cells expanded in Mesenchymal Stem Cell Growth Medium (MSCGM^TM^; Lonza) are termed 017-PC-O. (3) Cells expanded in Endothelial Cell Growth Medium MV2 (EGM MV2; PromoCell) are termed 017-PC-M. Each cell culture was fed once every 2 to 3 days and cells passaged at 80–90% confluency.

### Primary cell culture

Placental pericytes (Pl-PCs; PromoCell) and bone marrow derived mesenchymal stromal cells (BM-MSCs; PromoCell) were thawed and seeded into untreated/uncoated flasks. Pl-PCs were seeded at a density of 3–4 × 10^4^ cells per cm^2^ into 1 T-150 flask. Pl-PCs were cultured and expanded in Pericyte Growth Medium (PromoCell). BM-MSCs were seeded at a density of 4 × 10^4^ cells per cm^2^ into 1 T-150 flask. BM-MSCs were cultured and expanded in Mesenchymal Stem Cell Growth Medium (PromoCell). Both Pl-PC and BM-MSC cultures were fed once every 2–3 days and cells passaged at 80–90% confluency.

### Immunophenotype analysis

All cells were grown in corresponding media to at least 80% confluency. Cells were harvested and re-suspended in FC blocking buffer: 10% Fetal Bovine Serum (FBS) in Dulbecco’s Phosphate Buffered Saline (DPBS). Pre-labeled antibodies were diluted into FC blocking buffer. The following antibody conjugates labeled with APC, FITC or PE were used for antigen detection: CD31, CD34 and CD73 (Biolegend), CD146, CD105 (BD Biosciences); PDGFRβ, NG2 (R&D Systems); Tra-1-60, Oct-4 (Chemicon); CD133 (Dako). 5 × 10^5^ cells were incubated with 2 or fewer antibodies for 100 minutes at 4 °C. Cells were washed with FC blocking buffer and analyzed by flow cytometry (Accurri 6, BD Bioscience). Flow cytometry data was analyzed using FCS Express 4 (De Novo software).

### Cell differentiation

The osteogenic and adipogenic differentiation were performed in 24 well plates using StemPro^®^ Osteogenesis Differentiation Kit and StemPro Adipogenesis Differentiation Kit, respectively (Life Technologies). Control cultures were grown in 24 well plates using growth media as described above in cell culture and expansion. Control cultures and osteogenic differentiated cultures were stained with Alizarin Red S (Sigma). Control cultures and adipogenic differentiated cultures were stained with Oil Red O (Sigma). Brightfield images were taken at day 21 (osteogenesis) or day 14 (adipogenesis) (4X, Nikon TE2000).

### Tube formation assay

96 well plates were coated with Growth Factor Reduced Matrigel^TM^ (Corning); 50 μL Matrigel per well. Coated wells were equilibrated to room temperate for 20 minutes and then transferred to 37° C tissue culture incubator for 1 hour. Human umbilical vein endothelial cells (HUVECs) were pre-labeled with DiO dye (green; Life Technologies) and the test cells with Dil dye (red; Life Technologies) according to manufacturers’ recommended protocol. Cells were re-suspended and cultured in Endothelial Cell Basal Medium MV2 (PromoCell). Co-cultures contained a 20 to 1 ratio of HUVECs to test cells, seeded at 42,000 viable cells per well. HUVEC monocultures were seeded alone at 40,000 viable cells per well. Test cell monocultures were seeded alone at 18,750 or 2,000 viable cells per well (Figs. 5a and 6b). Brightfield and fluorescent images were taken beginning at 1 day and for up to 6 days to assess tube formation, tube stability and co-localization of HUVECs with test cells (4X, Nikon TE2000).

## Additional Information

**How to cite this article**: Greenwood-Goodwin, M. *et al*. A novel lineage restricted, pericyte-like cell line isolated from human embryonic stem cells. *Sci. Rep.* **6**, 24403; doi: 10.1038/srep24403 (2016).

## Supplementary Material

Supplementary Information

## Figures and Tables

**Table 1:** Differentiation potential and angiogenic support function hESC-derived perivascular progenitors and primary perivascular cells.

Cell Source	Cell Type	Adipogenic and OsteogenicDifferentiation Potential		Angiogenic Support Function Assayedby *In Vitro*Tube Formation	
		Oil Red O Staining	Alizarin Red Staining	Day 1	Day 6
Humanembryonicstem cells	017-PC-A	−	−	+	−
	017-PC-M	−	−	++	++
	017-PC-O	−	+	+	−
Bone Marrow	BM-MSC	+	+	+	−
Placental	Pl-PCs	−	+	+	−

Each cell type was evaluated for osteogenic and adipogenic potential; cells capable of differentiating down these linages are marked (**+**). Each cell type was evaluated for angiogenic support function as monitored by tube network formation for 6 days of cell culture (Figs 5, 6 and S6). Cells co-cultured with human umbilical vein endothelial cells showed (**−**), minimal (**+**) or extensive (**++**) tube network formation over multiple days.
