# Supplementary material for: A novel lineage restricted, pericyte-like cell line isolated from human embryonic stem cells
Source: Sci Rep. 2016 Apr 25;6:24403. doi: 10.1038/srep24403 (PMC4842973; doi:10.1038/srep24403)
Supplement: Supplementary Information [file srep24403-s1.pdf]

A novel lineage restricted, pericyte-like cell line isolated from human embryonic stem cells

Midori Greenwood-Goodwin, Ph.D.[1]*, Jiwei Yang, Ph.D.[2], Mohammad Hassanipour[1] and David Larocca, Ph.D.[1*]

[1] ReCyte Therapeutics, Inc., Alameda, CA 94502, USA.

[2] StemCells, Inc., Newark, CA 94560, USA.

* mgoodwin@biotimeinc.com, dlarocca@biotimeinc.com

MATERIALS AND METHODS

Image processing and quantification of tube formation assay

Tube formation assays were completed as described in the primary text. Phase-contrast images of HUVECs and test-cells in monoculture and co-culture, respectively, at 4X magnification were saved as TIFF files. Images were processed using Adobe Photoshop Elements 13 (Adobe Systems, San Jose, CA) and analyzed by the Angiogenesis Analyzer for ImageJ[1.]

Statistical analysis

Monoculture and co-cultures were analyzed in triplicate. 3 – 9 representative images of the tube formation assay were taken per condition (1 – 3 images per well). Data was expressed as mean ± standard error of the mean. Statistical significance was determined using one-way ANOVA with Tukey's post-hoc, $p < 0.05$ is considered statistically significant.

REFERENCES

1. Carpentier, G. Contribution: Angiogenesis Analyzer. *ImageJ News*, (2012).

FIGURES AND FIGURE LEGENDS

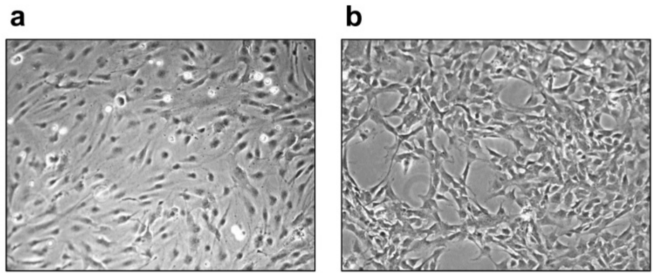

Supplementary Figure S1: Representative images of adherent cell morphologies following passaging of EBs initially seeded at low (a) or high (b) cell densities. EBs were seeded at low densities (2000 cells per well) or high densities (4000 cells per well). EBs were dispersed into single cell suspensions and seeded onto fibronectin-coated flasks. At confluency, adherent cell cultures display significantly different morphology despite identical culture media and substrate conditions. 4X magnification.

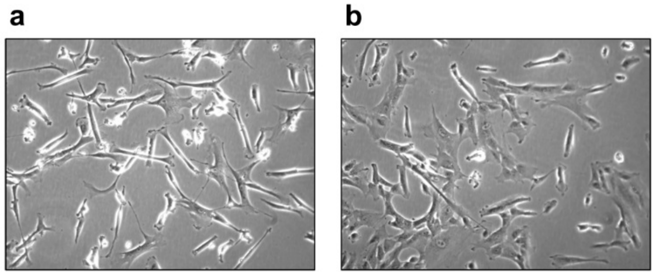

Supplementary Figure S2: Representative images of cell morphologies for bone marrow-derives mesenchymal stromal cells (MSCs; a) and 017-PC-O cells (b). Both cells were cultured in MSC-GM (PromoCell) for 3 days prior to imaging. 4X magnification.

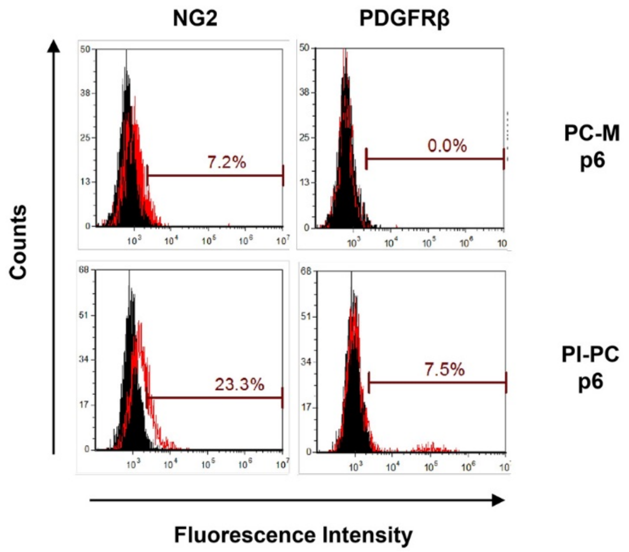

Supplementary Figure S3: Immunophenotyping for pericyte associate markers, NG2 and PDGFRβ. Representative flow cytometry analysis of 017-PC-M cells and placental pericytes (Pl-PCs) following in vitro expansion. The histograms shown here were gated on live cells using forward and side scatter profiles. The percentage of positive cells for specified markers (red) is shown here compared with isotype controls (black).

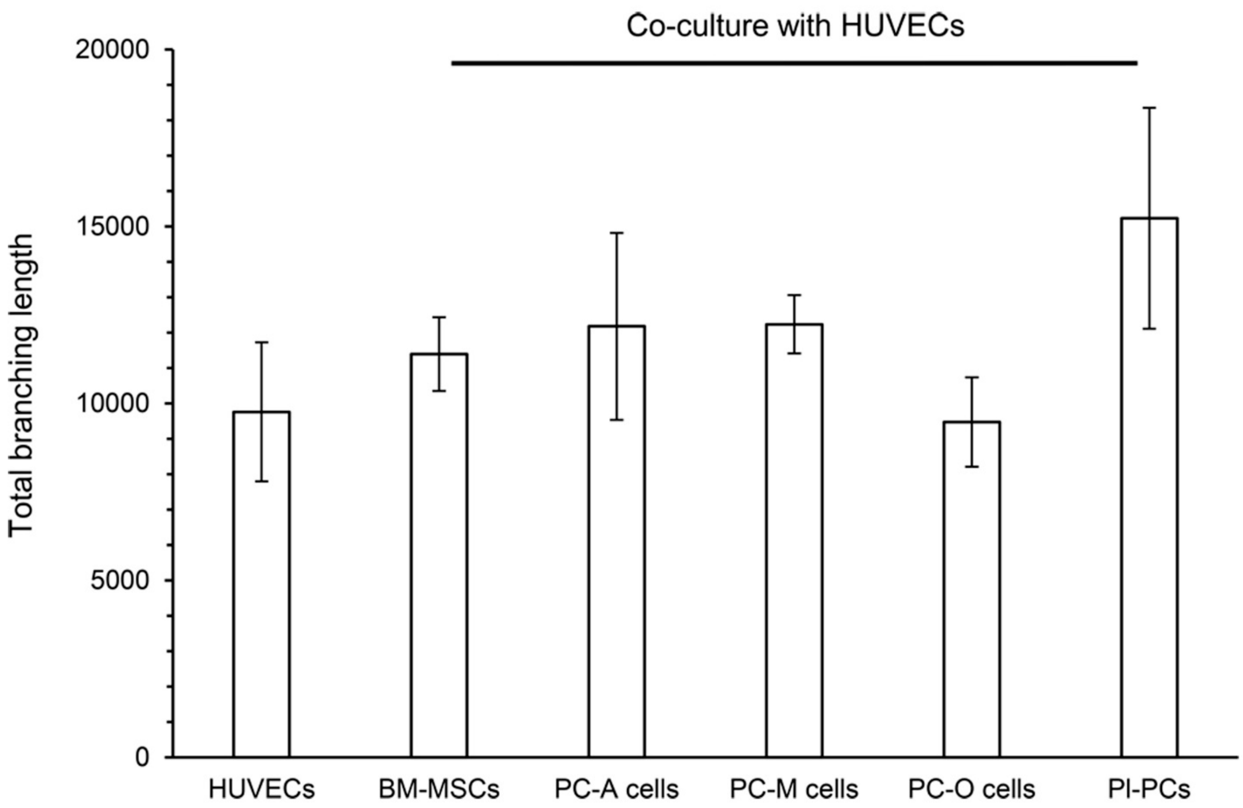

Supplementary Figure S4: Average total branching length at 1 day in co-cultures of human umbilical vein endothelial cells (HUVECs) with hESC-derived perivascular progenitors or primary cells. Co-cultures were seeded with 40,000 HUVECs and 2,000 hESC-derived perivascular progenitor or primary cells per well using 96 well plate format**.** Co-cultures were not statistically different across all conditions, including HUVECs in monoculture (far left). Triplicate images from HUVEC monoculture and co-cultures were used for Image J analysis using the Angiogenesis Analyzer plug-in; representative images for monocultures and co-cultures are shown in Figure 6A and Figure 5B, respectively. The difference in total branching length was not statistically significant ($p < 0.05$).

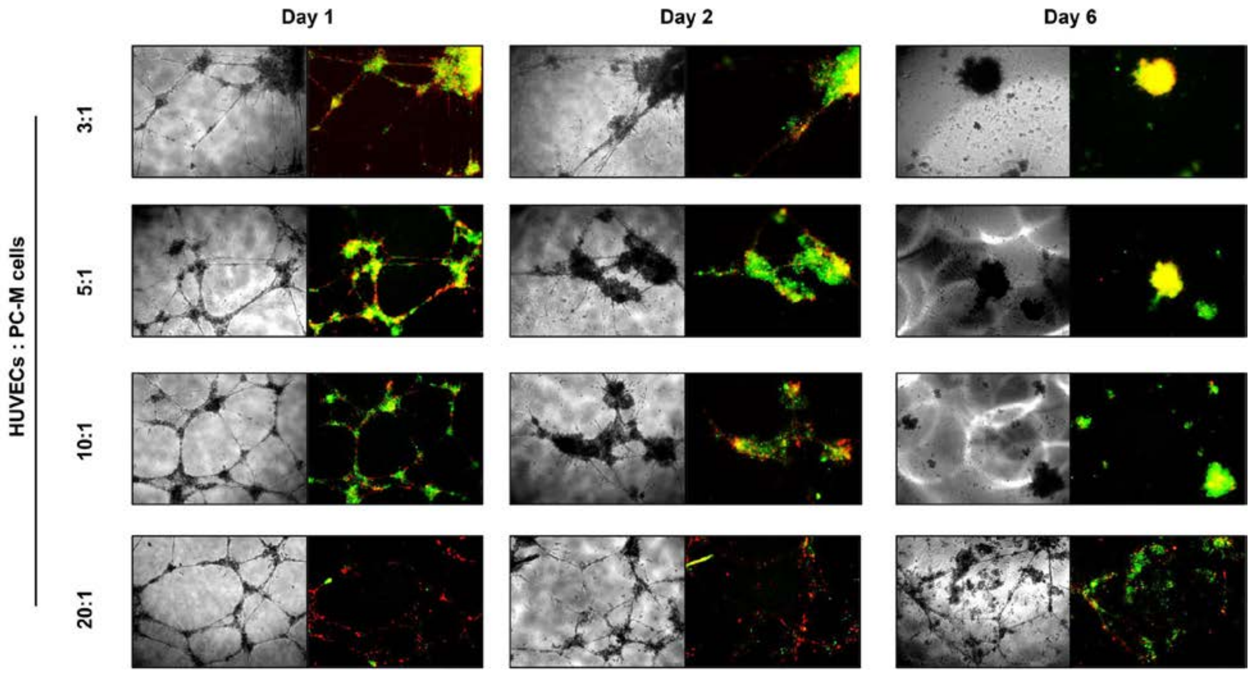

Supplementary Figure S5: Increasing seeding ratio of HUVECs to hESC-derived perivascular progenitor cell, 017-PC-M, improves tube network formation and stability in co-culture. Cells were seeded at 42,000 cells per well using 96 well plate format at a ratio of 3:1, 5:1, 10:1 and 20:1 HUVECs to 0-17-PC-M (PC-M) cells. All cultures were treated identically, without media exchange or the addition of exogenous growth factors. In co-culture, tube networks formed at a ratio of 20:1 HUVECs to PC-M cells showed less dense cell clusters on Day 1 compared with all other ratios and improved stability by Day 6.

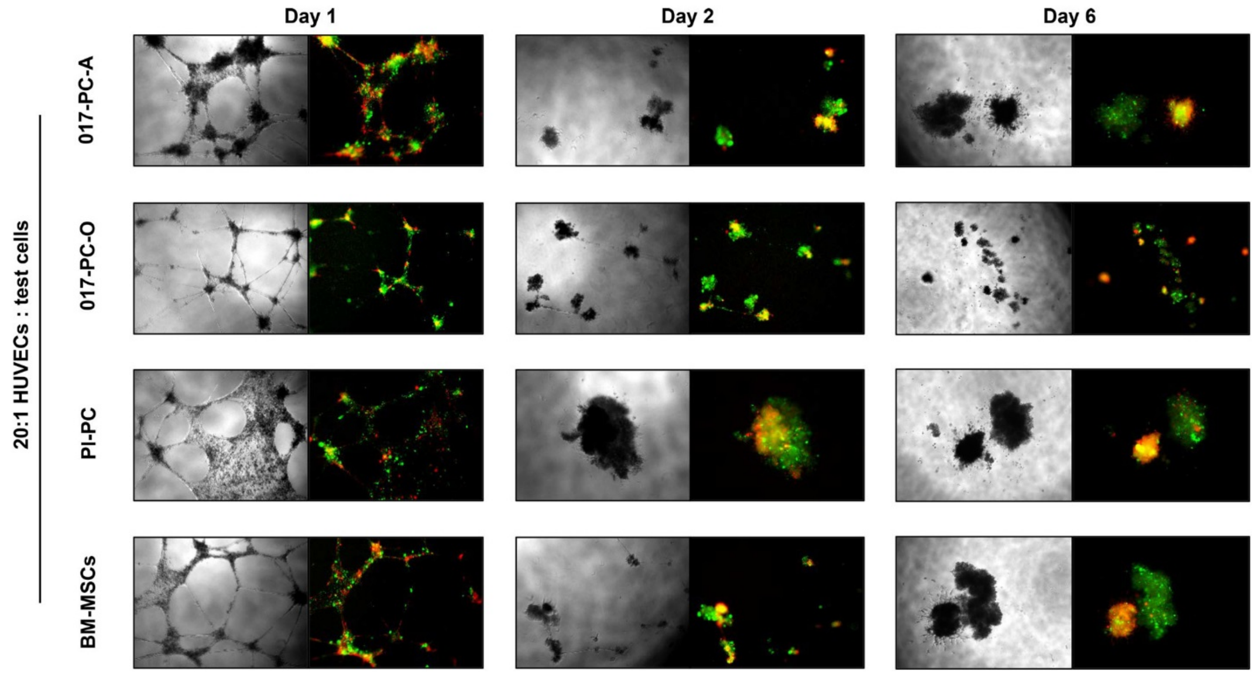

Supplementary Figure S6: hESC-derived perivascular progenitor cells, 017-PC-A and 017-PC-O, and primary cells show do not promote tube stability over multiple days. Cells were seeded at 42,000 cells per well using 96 well plate format at a ratio of 20:1 HUVECs to test cells. All cultures were treated identically, without media exchange or the addition of exogenous growth factors. In contrast to 017-PC-M cells (Figure 6), tube networks were significantly reduced by Day 2, with minimal tube-like structures observed. The presence of large cell clusters, as well as delocalization of HUVECs (green) and test cells (red) could be observed by Day 6.
